# Temporal retinal transcriptome and systems biology analysis identifies key pathways and hub genes in *Staphylococcus aureus* endophthalmitis

**DOI:** 10.1038/srep21502

**Published:** 2016-02-11

**Authors:** Deepa Rajamani, Pawan Kumar Singh, Bruce G. Rottmann, Natasha Singh, Manoj K. Bhasin, Ashok Kumar

**Affiliations:** 1 Kresge Eye Institute, Wayne State University, Detroit, MI; 2 Department of Anatomy and Cell Biology, Wayne State University, Detroit, MI; 3 Department of Immunology and Microbiology, Wayne State University, Detroit, MI; 4 BIDMC Genomics, Proteomics, Bioinformatics and Systems Biology Center, Beth Israel Deaconess Medical Center, Boston, MA; 5 Department of Medicine, Division of Interdisciplinary Medicine and Biotechnology, Beth Israel Deaconess Medical Center, Harvard Medical School, Boston, MA 02115, USA

## Abstract

Bacterial endophthalmitis remains a devastating inflammatory condition associated with permanent vision loss. Hence, assessing the host response in this disease may provide new targets for intervention. Using a mouse model of *Staphylococcus aureus* (SA) endophthalmitis and performing retinal transcriptome analysis, we discovered progressive changes in the expression of 1,234 genes. Gene ontology (GO) and pathway analyses revealed the major pathways impacted in endophthalmitis includes: metabolism, inflammatory/immune, antimicrobial, cell trafficking, and lipid biosynthesis. Among the immune/inflammation pathways, JAK/Stat and IL-17A signaling were the most significantly affected. Interactive network-based analyses identified 13 focus hub genes (IL-6, IL-1β, CXCL2, STAT3, NUPR1, Jun, CSF1, CYR61, CEBPB, IGF-1, EGFR1, SPP1, and TGM2) within these important pathways. The expression of hub genes confirmed by qRT-PCR, ELISA (IL-6, IL-1β, and CXCL2), and Western blot or immunostaining (CEBP, STAT3, NUPR1, and IGF1) showed strong correlation with transcriptome data. Since TLR2 plays an important role in SA endophthalmitis, counter regulation analysis of TLR2 ligand pretreated retina or the use of retinas from TLR2 knockout mice showed the down-regulation of inflammatory regulatory genes. Collectively, our study provides, for the first time, a comprehensive analysis of the transcriptomic response and identifies key pathways regulating retinal innate responses in staphylococcal endophthalmitis.

Despite several advances in ocular surgeries, including sutureless and minimally invasive procedures, infectious endophthalmitis remains one of the most concerning postsurgical complications due to its ability to cause blindness^1^,^2^,^3^. The incidence of endophthalmitis varies with the surgical procedure performed, but it is most likely to develop following cataract surgery/intraocular lens implantation, with the incidence ranging from 0.07% to 0.4%^4^,^5^. This reported incidence represents a significant public health problem, as >3.4 million cataract surgeries are performed in the United States annually, and over 19.5 million performed worldwide^6^,^7^. Moreover, the incidence of endophthalmitis is expected to grow in the near future due to the increased use of multiple intravitreal injections (IVTs) for the treatment of age-related macular degeneration (AMD) and diabetic retinopathy (DR)^8^,^9^.

The visual outcome following endophthalmitis largely depends upon the virulence potential of the infecting pathogen and the dynamic interplay between the host and the pathogen^10^,^11^. For example, eye infected with *Staphylococcus epidermidis* exhibit mild acute inflammatory response and the infection is resolved quickly, with restoration of visual acuity close to normal^12^,^13^,^14^. In contrast, inoculation of the eye with the other Gram-positive bacteria, such as *Bacillus cereus*^15^,^16^,^17^,^18^ and *S. aureus*^3^,^19^,^20^,^21^,^22^,^23^, results in severe intraocular inflammation, retina tissue damage, and loss of vision within 24 to 96 h post-infection, respectively. However, regardless of the nature of the pathogens, the common host response to an infection, including endophthalmitis, is the generation of inflammatory responses. In the past decade, extensive studies from our laboratory and others have provided a considerable wealth of information on how innate responses are initiated in the retina during endophthalmitis.

One of the cardinal steps in initiating an innate immune response is pathogen recognition by specific receptors present on innate immune cells^24^. The best characterized class of receptors which the host employs are called the Toll-like receptors (TLRs)^25^, whose discovery has opened up a wide range of therapeutic opportunities for various infectious and inflammatory diseases^26^. Consequently, the role of TLRs has also been well-established in ocular infections^27^, including bacterial endophthalmitis^19^,^20^,^28^,^29^,^30^,^31^. We previously reported that TLR2 plays an important role in innate immune responses in SA endophthalmitis^2^,^19^,^31^,^32^,^33^. Moreover, we showed that TLR2-activation prior to SA infection attenuated the development of endophthalmitis in a mouse model^20^. Furthermore, TLR2 ligand Pam3Cys (Pam3) pretreatment was found to reduce the inflammatory response and enhance the phagocytic activity of microglial cells following SA challenge^19^. How Pam3 pretreatment modulates global retinal innate responses in bacterial endophthalmitis is not known. Thus, considering the complex nature of host-pathogen interactions, we performed genome wide transcriptome analysis of SA-infected retinal tissue. Here, we established a molecular signature of staphylococcal endophthalmitis, revealed several key pathways, and identified 13 master regulator genes whose expression is modulated by TLR2 signaling.

## Results

### SA infection leads to temporal qualitative and quantitative gene expression alterations

**Figure 1:**
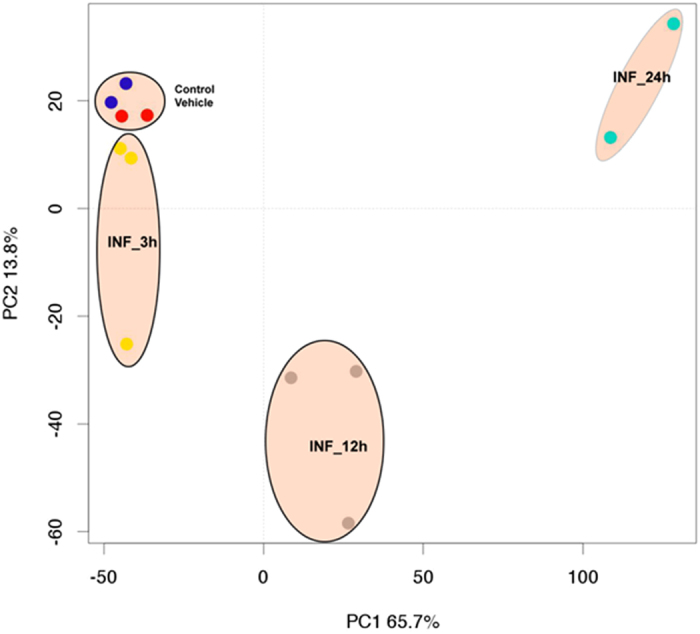
Principal Component Analysis (PCA) of normalized expression data obtained from controls and SA infected samples. The first component with the highest variance (65.7%) is on the X-axis separating 12 and 24 h post-infection samples from control samples and 3h post- infection samples. The second component, with the second highest variance (13.8%), is on the Y-axis, depicting a maximum variation of the 12 h post-infection samples from rest of samples. The biological replicates from control, 3 h, 12 h, and 24 h samples formed separate clusters on the PCA plot, indicating transcriptional differences among different groups.

Principal component analysis (PCA) demonstrated that 12 h and 24 h post-infection samples are separated from controls and 3 h post-infection samples along Principal Component 1 (PC1), which accounts for 65.7% of the variance in transcriptome data. This demonstrates that transcriptional differences were greater at later time points (e.g. 12 h, 24 h) of SA infection, as compared to an early time point, 3 h post-infection, and compared to uninfected controls. This also suggests that SA infection initially results in a potent minimal response, manifesting as an early qualitative and quantitative changes in gene transcription, which grows and spreads exponentially over time. The analysis also revealed distinctive clusters of samples consistent with the biological groups on the basis of the transcriptional profiles (Fig. 1).

### Characterization of changes in transcriptional landscape due to SA infection

After preprocessing, we conducted group-wise comparisons using t-tests and fold change to identify differentially expressed genes at each time point after SA infection compared to control/vehicle samples. The group comparison identified the sets of genes that were significantly differentially expressed at each time point compared to vehicle and non-infection samples (Fig. S1A). All time points post-infection – (early (3 h), mid (12 h), and later (24 h) – evoked significant gene expression changes as compared to vehicle and uninfected control samples (Fig. S1A). The analysis identified a large number of differentially expressed genes 24 h post infection versus 3 h post infection, indicating more pronounced transcriptional changes at later time points. Overall, the analysis identified 1,234 genes that are differentially expressed at multiple time points after infection and might be strongly associated with the initiation and progression of infection (Fig. S1B). Out of 1,234 genes, 133 genes were consistently differentially expressed at all three-time points post SA infection as compared to control/vehicle samples. These genes are significantly associated with the response to DNA damage, cell death and apoptosis, and the regulation of transcription and RNA splicing (Fig. S1C).

### SA infection elicits constitutive and temporal patterns of differential gene expression: Self-Organizing Map (SOM) Analysis

**Figure 2:**
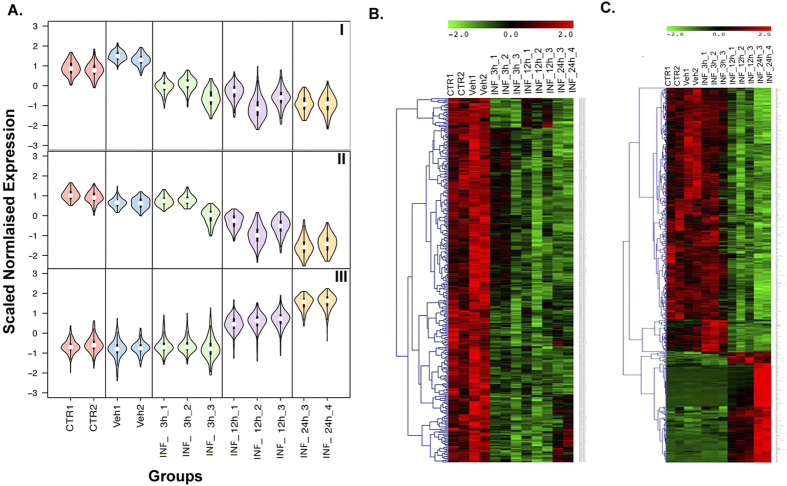
Significant transcriptional changes induced due to infection. (**A**) Self-organizing map (SOM) analysis of 1,234 genes commonly differentially expressed among different post-infection time points compared to controls. The SOM maps were clustered into ‘constitutive’ (similar expression changes at 3 h, 12 h, and 24 h) and ‘temporal’ (progressive expression changes at 3 h, 12 h, and 24 h) expression changes on the basis of structure of expression pattern. The figure shows violin plots of selected 3 maps depicting constitutive down-regulation (I), temporal down-regulation (II) and temporal up-regulation (III) patterns. Each cluster represents a set of genes that depict similar expression patterns and are biologically linked to a specific function. The X-axis represents different time points and groups, and the Y-axis represents gene expression on pseudoscale from −3 to +3. (**B**) Heatmap of constitutive genes depicting similar expression changes at 3 h, 12 h, and 24 h post-infection. (**C**) Heatmap of temporal genes depicting progressive expression changes from 3 h, 12 h, and 24 h post-infection. Columns represent samples, and rows represent genes. Gene expression levels are shown on a pseudocolor scale (−1 to 1), with red denoting a high expression level and green denoting a low expression level.

To identify gene expression patterns, we performed SOM analysis on 1,234 genes that were differentially expressed at multiple time points after SA infection, as identified by the group comparison analysis above^34^. Initially, differentially expressed genes were partitioned to 40 SOM patterns with different expression structures. Based on their similarity in gene expression patterns, we selected and merged these patterns into 4 distinct categories of related patterns. These 4 categories reflect the following patterns of gene expression changes: 1. “constitutive” upregulation; 2. “constitutive” downregulation; 3. “temporal” upregulation; and 4. “temporal” downregulation (Fig. 2). Overall, SA infection results in temporal expression changes, as well as a general shift in the baseline expression of many important genes. We defined constitutive downregulation patterns as those for which gene expression levels were consistently lower in all post SA infection samples compared to both controls (Fig. 2A, Panel I). These genes depict an overall shift in gene expression following infection, independent of time of SA infection (early/mid/late), and might be responsible for sustaining the infection (Fig. 2B). We also observed a temporal downregulation pattern, where genes had the highest gene expression values at 3 h, with lower levels of expression at 12 h and the lowest levels at 24 h (Fig. 2A, Panel II). We defined a temporal upregulation pattern as the genes that showed a gradual increase in expression according to the length of infection. In other words, gene expression changes were low of insignificant at 3 h, higher 12 h, and the highest 24 h post-infection (Fig. 2A, Panel III). A heat map of temporally dysregulated genes after SA infection is shown in Fig. 2C that depict changes in gene expression that are highly correlated with the time of SA infection and set of genes that may be responsible for initiating, sustaining, and spreading SA infection.

### SA infection dysregulates biological gene sets and functions related to metabolism, cell cycle, apoptosis, and kinase activity

To gain insight into the functional consequences of altered gene expression due to SA infection, we employed the Database for Annotation, Visualization and Integrated Discovery (DAVID). The constitutively regulated genes that show significant enrichment in clusters of the gene ontology (GO) categories related to metabolism (e.g. Nucleotide, Macromolecules), RNA splicing, and protein ubiquitination are shown in Fig. S2A. The most highly enriched clusters of the GO categories regulated temporally by SA infection include those related to programed cell death, the inflammatory response, carbohydrate binding, cell activation, the immune response, and actin binding related genes (Fig. S2B). Further functional analysis on genes temporally associated with SA infection showed upregulation of genes linked to cellular movement, immune cells trafficking, inflammatory response, and cellular growth and proliferation, as well as downregulation of genes linked to cell death and survival and tissue morphology (Fig. S2C).

### SA infection temporally activated multiple immune response and inflammation-related pathways

**Figure 3:**
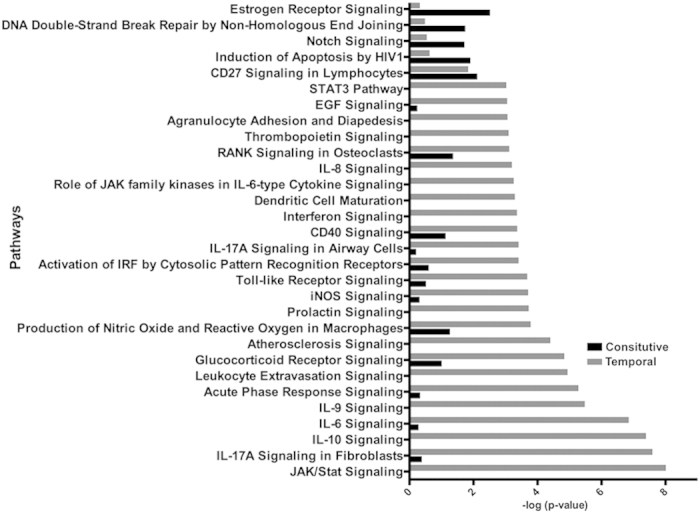
Comparison of affected pathways between constitutively and temporally altered genes post-infection. Pathways significantly enriched in constitutive versus temporal post-infection altered genes. Pathways affected by constitutively altered genes are shown as black bars, while those affected by temporally altered genes are shown as grey bars.

As a complementary approach, pathway enrichment analysis was performed on genes with temporal transcriptional changes following SA infection. Pathways enrichment was assessed using Ingenuity Pathway Analysis (IPA) tools. This IPA demonstrated significant enrichment of temporally altered genes involved in numerous inflammatory response and immune system-related pathways (Fig. 3 and Table 1). In keeping with the inflammatory properties of SA infection, JAK/Stat Signaling, IL-17 A, IL-10, IL-6, and IL-9 acute phase signaling pathway, interferon signaling, glucocorticoid receptor signaling, iNOS signaling, Toll-like receptor signaling, CD40 signaling, and the STAT3 pathways were marked by an overall up-regulation of relevant transcripts. Additionally, constitutively altered genes depicted significant enrichment in CD27 signaling, estrogen receptor signaling, protein ubiquitination, AMPK, and apoptosis-related (TNFR-1) pathways (Table 1).

### Systems biology analysis of SA infection transcriptome changes identified activation of immune and inflammatory responses as the key master regulator

**Figure 4:**
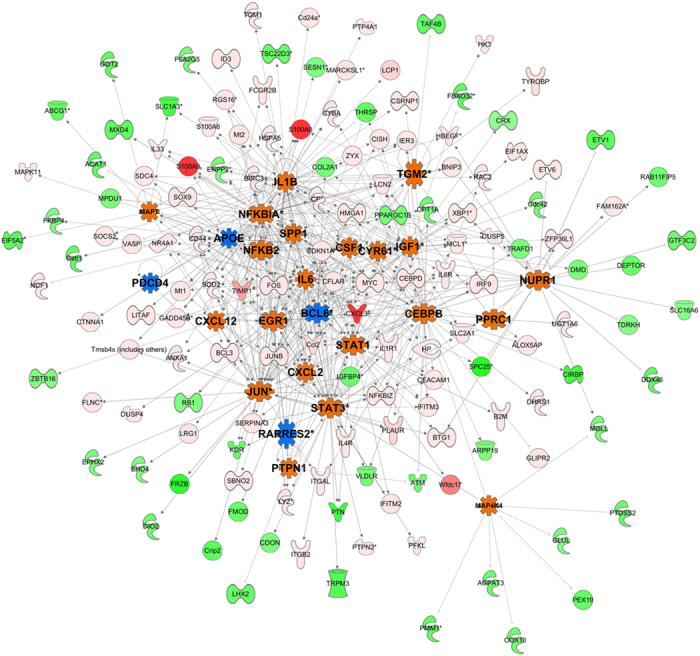
Interactive network of the regulatory molecules and their down-stream targets that are significantly activated or inhibited during the temporal post-infection phase. Top regulatory genes were selected on the basis of enrichment score, as derived from the dysregulation of target molecules and their fold changes due to infection. In this network, activated and inhibited regulatory molecules are shown with orange and blue color, respectively. The regulatory molecules include the activation of multiple key inflammation related molecules, including NFKB2, IL-1β, CXCL2, and JUN. Also, in this network, each node represents a gene and each edge represents an interaction. The up-regulated and down-regulated target genes are shown with shades of red and green colors, respectively.

To further understand the molecular mechanisms of SA infection, we performed systems biology analysis on constitutively or temporally altered transcripts. The systems biology analysis was performed using a master regulator approach that assists in identifying the key transcriptional regulators that might be responsible for pathophysiology. The regulatory analysis depicted significant activation of multiple major immune and inflammation regulators, including STAT1, STAT3, IL-6, NFKB2, JUN, SPP1, IL-1β, CSF1, CXCL2, IGF1, CEBPB, and PTPN1 (Fig. 4). The systems biology analysis clearly depicts activation of the innate immune response of the host via activation of the key transcriptional regulator NF-κB, as well as interferons and cytokines.

### Molecular Effects of Pam3 Treatment to blunt SA infection

We previously reported that TLR2 activation induces protective responses in SA endophthalmitis^19^,^20^. To understand the molecular effects of TLR2 ligand (Pam3) treatment, we performed whole genome transcriptome profiling on Pam3 treated (PAM3) and PAM3–pretreated and SA infected (Pam3+SA) samples. The transcriptome analysis identified 406 and 1,090 differentially expressed genes in Pam3 and Pam3+SA treated samples, respectively, as compared to controls (Fig. S3A). The analysis identified 85 transcripts that are commonly differentially expressed in Pam3 and PAM3+SA treated samples (Fig. S3B). These common transcripts are significantly linked to biological processes involved in cellular development, cellular growth and proliferation, and cellular morphology (Fig. S3C). Further pathway enrichment analysis on these transcripts showed a significant overrepresentation in protein ubiquitination, DNA methylation, lactose degradation, and apoptosis signaling pathways (Fig. S3D).

### Counter regulation of SA infection mediated transcriptome changes by PAM3

**Figure 5:**
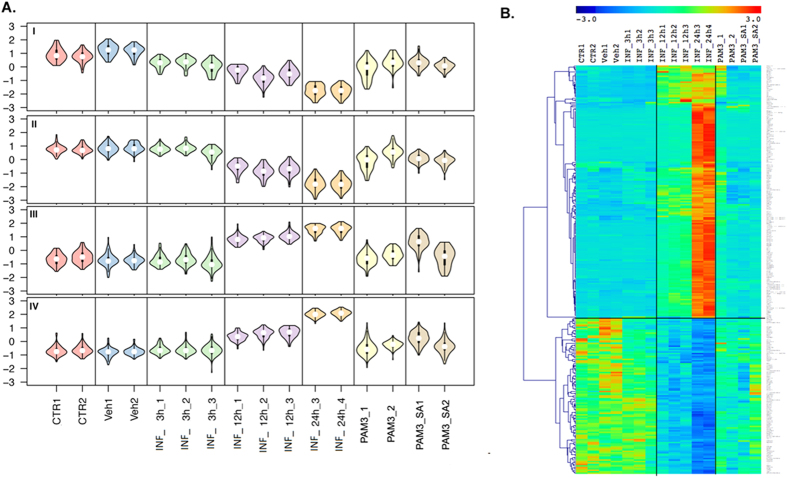
Counter regulation of gene expression by PAM3 pre-treatment, as determined by transcriptome profile comparison of temporal samples with and without PAM treatment prior to infection. (**A**) SOM maps representing infection-related gene clusters that were significantly counter regulated by PAM3 pre-treatment. PAM3 pre-treatment counter regulated 280 genes out of the approximately 1,200 genes altered due to infection. (**B**) Heatmap of genes that are significantly counter regulated due to PAM3 pre-treatment. Columns represent samples, and rows represent genes. Gene expression levels are shown on a rainbow pseudocolor scale (−3 to 3), with red denoting high expression levels and blue denoting low expression levels.

The PAM3-mediated development of a microenvironment suitable for combatting SA infection was studied by PAM3 counter regulation analysis. SOM counter analysis was performed on 1,234 differentially expressed genes induced by SA infection to identify the effects of PAM3 treatment on these genes. Counter-regulation means that PAM3 treatment down-regulates genes that are up-regulated in SA infection vs. controls and vice versa. SOM clustering of differentially expressed transcripts post-infection to 10 bins was performed to identify transcripts that are counter regulated by PAM3 treatment. Bins of transcripts manifesting different degrees of counter regulation were identified and further filtered on the basis of magnitude of counter regulation. The final list of counter regulated genes was generated by considering genes that are counter regulated by a magnitude of fold change (FC) > 1.2–1.5 on treatment, as compared to their expression 24 h post-SA infection. The analysis identified genes that were temporally regulated by SA infection and counter regulated by PAM3 treatment (Fig. 5A). Figure 5A shows violin plots which reveal two major themes in gene expression pattern counter regulation by PAM3. Panels I and II of Fig. 5 shows the up-regulation of temporally down-regulated genes due to SA infection (with slight variations in the temporal gene expression pattern) upon treatment with PAM3, while Panels III and IV show the down-regulation of temporally up-regulated genes due to SA infection upon PAM3 treatment. Furthermore, PAM3 pre-treatment creates a microenvironment by counter regulating the SA infection signature, which is maintained 24 h post-secondary SA infection after PAM3 treatment (Fig. 5A; last time point shown). The heat map of the 280 SA infection signature genes clearly shows this counter regulation pattern by PAM3 treatment (Fig. 5B).

**Figure 6:**
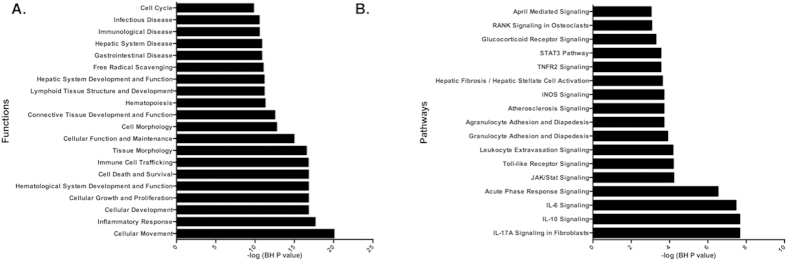
Functional and Pathway enrichment analysis of genes significantly counter regulated by PAM3 pre-treatment. (**A**) Functional enrichment analysis depicting the counter regulation of multiple functional categories related to the immune and inflammatory response, as well as cell death and survival. (**B**) Pathway enrichment analysis depicting the counter regulation of multiple inflammatory and cell adhesion response related pathways, including IL-10 signaling, IL-6 signaling, JAK/STAT signaling, TNFR2 signaling, and Toll-like receptor signaling. The significance of the effect of PAM3 pre-treatment on functional and pathway categories was determined using multiple test corrected Fisher’s exact test *p* values.

The SA infection signature genes that were counter regulated by PAM3 treatment were further investigated for functional and pathway enrichment using Ingenuity Pathway Analysis (IPA) tools. The functional groups most significantly enriched related to two major categories, namely, inflammatory processes and basic cellular functions. The top inflammatory functional categories included cellular movement, inflammatory response, immune cell trafficking, immunological disease and infectious disease, and the top functions associated with general cellular functioning included cellular development, cell growth and proliferation, hematological system development and function, cell death and survival, and cellular morphology (Fig. 6A). Furthermore, pathways enrichment analysis on PAM3 counter regulated genes depicted a significant enrichment in multiple inflammatory pathways like IL-17 A signaling in fibroblasts, IL-10 signaling, IL-6 signaling, acute-phase response signaling, JAK/STAT signaling, and Toll-like receptor signaling (Fig. 6B).

### PAM3 treatment results in significant suppression of master regulators of SA infection

**Figure 7:**
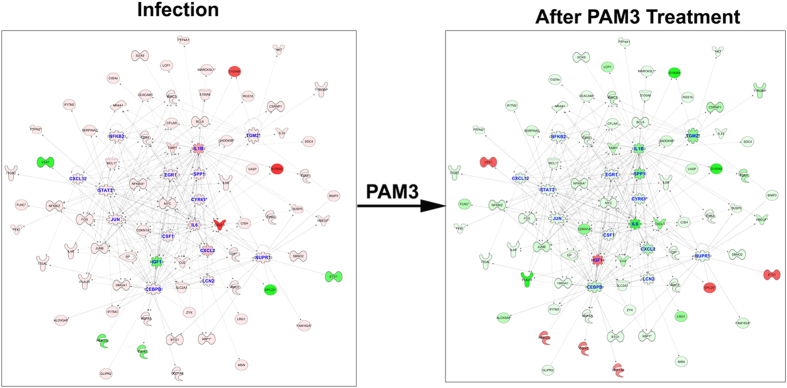
Master regulatory molecules counter regulated by PAM3 Pre-treatment to improve the outcome following infection.

Systems biology analysis of genes associated with the PAM3 counter regulation signature further identified the major networks formed by these genes and predicted the master regulators and there up- or down-regulation based on the expression patterns of the network genes. It is very evident from the analysis that the magnitude of differential expression and its directionality are reversed for these PAM3 counter regulation signature genes before and after PAM3 treatment (Fig. 7). The hub genes like IL-6, JUN, and CXCL2 are predicted to be up-regulated before PAM3 treatment and are predicted to be down-regulated after PAM3 treatment based on the observed expression levels of the PAM3 counter regulatory signature genes. Similarly, IGF1 is predicted to be down-regulated master regulator before PAM3 treatment and up-regulated after treatment.

### Validation of SA infection master regulators at mRNA and protein levels

**Figure 8:**
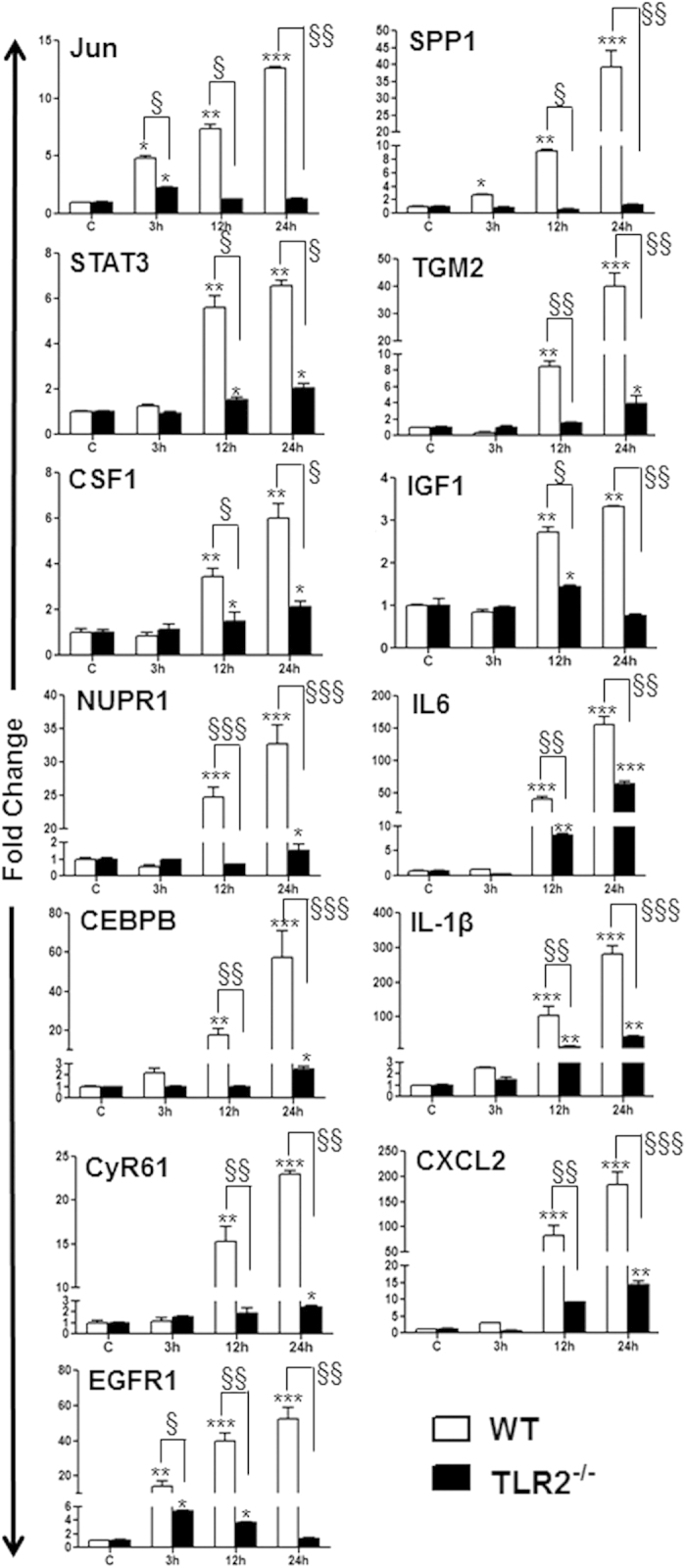
qRT-PCR analysis of regulatory molecules for validation of gene expression data. C57BL/6 or TLR2^−/−^ (B6 background) mice were intravitreally injected with *S. aureus* (SA) for the indicated time points. Total RNA was extracted, reverse transcribed, and subjected to qRT-PCR using specific primers for thirteen master regulator genes (IGF1, Jun, STAT3, NUPR1, CEBPB, CSF1, CyR61, EGFR1, SPP1, TGM2, IL-6, IL-1β, and CXCL2) with glyceraldehyde 3-phosphate dehydrogenase (GAPDH) as the control. Modulations of gene expression were expressed as relative fold changes with respect to the GAPDH control. Statistical analysis was performed using one-way ANOVA (*) and Student’s t-test (^§^) for comparisons of control versus stimulated mice over time, and C57BL/6 vs. TLR2^−/−^ mice, respectively. Data points and error bars represent the mean ± SD of triplicates from three independent experiments. (*^§^*P* < 0.05; **^§§^*P* < 0.005; ***^§§§^*P* < 0.0005).

**Figure 9:**
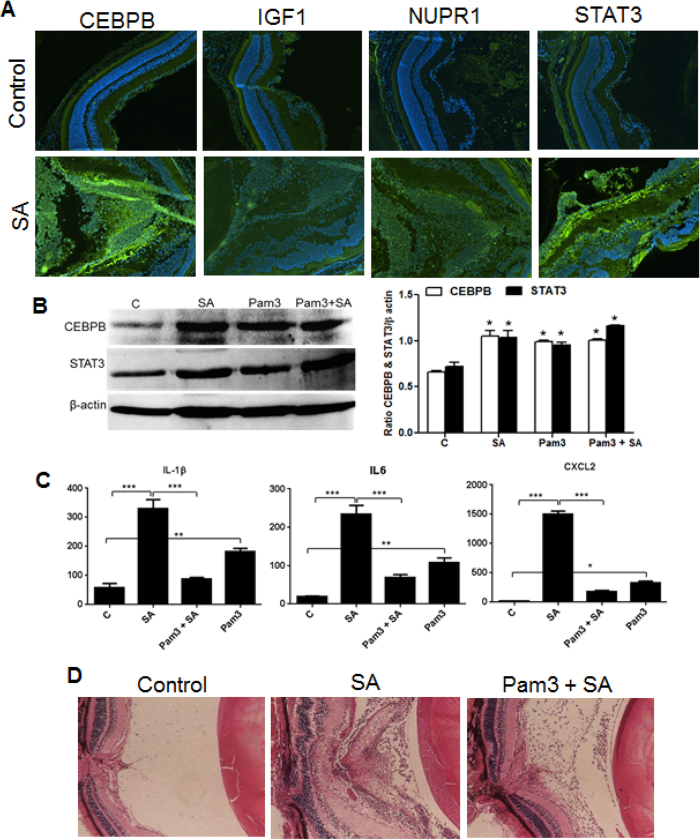
Analysis of regulatory molecules at the protein level. C57BL/6 mice were given intravitreal injections of PBS (control) or SA/Pam3. After 24 h, enucleated eyes were either embedded in OCT or fixed in paraffin. Cryosections were subjected to immunostaining (**A**), as described in material and methods. From a separate group, eyes were enucleated and lysates were made in PBS using a tissue lyser. Retinal lysates were subjected to Western blotting to validate protein expression (**C**, left panel). The band intensities were quantitated by densitometric analysis using Image J and presented as bar graph using β-actin as control (**C** right panel). Protein levels of inflammatory cytokines were assessed by ELISA (**B**). Paraffin embedded eyes were subjected to H&E staining for histological analysis (**D**). (*P < 0.05, **P < 0.005, ***P < 0.0005 ANOVA).

To validate the microarray data, the expression profile of all 13 master regulator genes (IGF1, Jun, STAT3, NUPR1, CEBPB, CSF1, CyR61, EGFR1, SPP1, TGM2, IL-6, IL-1β, and CXCL2) was confirmed by qRT PCR in both WT and TLR2 KO mouse retina. Our data revealed that TLR2 deficiency resulted in the downregulation of the hub genes (Fig. 8). The overlay of qRT-PCR and microarray expression profiles of the hub genes in WT mouse retinas showed strong correlation (Fig. S4). To determine whether the master regulator genes are expressed at the protein level and whether their expression is modulated by Pam3 treatment, we performed IHC, Western blot, and ELISA assays (Fig. 9). To this end, our data showed that SA strongly induced the expression of CEBPB and STAT3 in the retina, whereas the expression of NUPR1 and IGF1 was relatively low, as assessed by IHC (Fig. 9A). The induced expression of CEBPB and STAT3 was further confirmed by Western blot analysis (Fig. 9B). The protein levels of the master regulatory cytokines IL-1β and IL-6 and chemokine CXCL2 were accessed by ELISA. As expected, compared to uninfected controls, SA infection resulted in the increased expression of IL-1β, IL-6, and CXCL2 and their levels were significantly lower in Pam3 pretreated retinal tissue (Fig. 9C). To ascertain whether Pam3 pretreatment protected retinal tissue from SA endophthalmitis, H&E staining was performed. As anticipated, SA causes cellular infiltration and retinal folding/damage; however, these pathological changes were abolished in Pam3-pretreated eyes (Fig. 9D).

## Discussion

Endophthalmitis is an inflammatory disease caused by intraocular microbial infections, which result in blindness in some patients. Severe vision loss in endophthalmitis is the result of retinal damage caused by pathogen derived virulence factors (e.g., toxins) and excessive host inflammatory responses^35^,^36^. Although the mechanisms underlying the initiation and resolution of intraocular inflammation in bacterial endophthalmitis are not fully understood, our previous studies implicated the role of TLRs^26^. We demonstrated that, once bacteria gain access to the inside the eye, retinal residential cells (microglia and Müller glia) become activated and initiate retinal innate responses^2^,^19^,^32^,^33^ due, at least in part, to TLR2 signaling in case of SA infection^26^,^37^. We also demonstrated that TLR2 ligand (Pam3Cys) pretreatment protected mice from SA endophthalmitis, which is, in part, mediated by reduced inflammatory response and the increased production of antimicrobial peptides^20^. To further delineate the protective mechanisms evoked by TLR2 engagement and to establish a molecular signature of staphylococcal endophthalmitis, in this study, we determined the differential gene expression in uninfected, SA-infected, and Pam3 pretreated and SA-infected mouse retinas.

Transcriptome analysis of retinal tissue at 3 h, 12 h, and 24 h after infection provides deep insight into the gene, pathway, and biological networks that are perturbed during different phases of infection. Here, we established that SA infection results in either constitutive or temporal dysregulation of host genes. The constitutive patterns consist of genes that are immediately dysregulated upon SA infection and remain dysregulated throughout the infection. It is possible that these genes may be vital to the survival of the pathogen. Functional analysis of these genes depicted significant enrichment in metabolic and cellular maintenance machinery and apoptosis-related biological processes. The changes in gene expression demonstrated significant association with estrogen signaling, protein ubiquitination, and AMPK signaling. It is well known that protein ubiquitination plays a critical role in the initiation and termination of innate response signal transduction pathways, such as nuclear factor κB (NF-κB) pathway, which evokes a wide range of host inflammatory responses to inhibit bacterial growth^38^. In addition, ubiquitination can induce the host cell to kill pathogens via proteasome and phagolysosome-mediated degradation pathways. Some pathogens can hijack ubiquitination pathways to evade the host immune response^39^. This phenomenon is well demonstrated among Gram-negative bacteria such as *Salmonella*, *Shigella*, and *Pseudomonas* spp., where the type III and type IV secretion systems (T3SSs and T4SSs) are particularly involved in targeting host ubiquitin systems^40^. Although *S. aureus* produces a variety of virulence factors, including toxins, to our knowledge their role in disrupting ubiquitin pathways has not been reported. Our study, showing a constitutive dysregulation of ubiquitination pathways (e.g. UBE3A) in SA-infected retinal tissue, indicates that SA may utilize these mechanisms for survival in the eye and that the activation of specific ubiquitin mechanisms may promote bacterial clearance. Similarly, SA endophthalmitis was found to alter the expression of genes/pathways involved in cellular metabolism, including increased fatty acid synthesis and reduced energy/ATP generating signaling pathways, such as AMPK signaling (Table 1). Indeed, we confirmed these transcriptome findings and evaluated the role of AMP-signaling in SA endophthalmitis. Our data (manuscript under review) revealed that SA infection resulted in the downregulation of AMPK activity, an enzyme that plays a key role in cellular energy homeostasis. This reduction in AMPK activity (phosphorylation) coincided with increased inflammation and the restoration of AMPK phosphorylation using a specific activator, AICAR, attenuated inflammation in SA-infected eyes. Together, these observations support the biological significance of the results reported in the current study.

The genes with temporal transcriptome changes demonstrated that SA infection targeted multiple pathways and biological processes that were central to immune and inflammatory responses and cellular growth and proliferation, as expected, but also revealed novel pathways impacted by SA infection, such as the glucocorticoid receptor signaling, RANK/RANKL signaling, protein ubiquitination, and Notch signaling. Pathway analysis revealed an overall activation of multiple immune and inflammation pathways, including JAK/STAT signaling, interleukin pathways (e.g., IL-10, IL-6, IL-9, IL-8, IL-17 A), interferon signaling, and Toll-like receptor (TLR) signaling. The induced expression of genes/molecules involved in TLR signaling was expected, as extensive studies from our^2^,^19^,^20^,^26^,^32^,^41^ and other^28^,^30^,^42^,^43^ laboratories have demonstrated an essential role of TLRs in retinal innate responses in bacterial endophthalmitis. Interestingly, among the five prominent signaling pathways (Table 1) with temporal changes, IL-17 A and JAK/STAT were the most significantly affected. The role of these pathways in the pathogenesis of bacterial endophthalmitis is yet to be elucidated. Previous studies have shown that IL-17 induces protective innate immunity against SA infection in the skin^44^. Moreover, IL-1, TLR2, and IL-23 were found to promote neutrophil recruitment to the SA-infected skin by inducing IL-17 production in γδT cells^44^. IL-17 deficiency diminished lung defenses against SA by reducing the production of antimicrobial peptides and neutrophil-recruiting chemokines^45^. The role of IL-17 has also been elucidated in fungal keratitis where a subset of neutrophils was assigned as the cellular source of IL-17^46^,^47^. Similarly, a transcriptome study of SA-infected corneal epithelium revealed the induced expression of CCL20^48^, a strong Th17 chemoattractant^49^. Given the strong relationship of IL-17 and SA infection, it would be interesting to investigate the role of IL-17 signaling in SA endophthalmitis by inhibition or neutralization studies and confirm the transcriptome findings at the protein level.

Systems biology analysis of the SA infection signature assisted in generating a global picture of the crosstalk between the pathways/genes involved in pathophysiology and to highlight the master regulators and key processes that are most critical for SA endophthalmitis. The systems biology analysis identified multiple immune system and inflammation molecules (e.g., STAT3, STAT1, CEBPB, JUN, IGF1, SPP1, NFKB2, IL-1β, and CSF1) as master regulators that are activated upon infection. On the other hand, genes linked to cell proliferation and apoptosis (e.g., BCL6, APOE, PDCD4, and RARES2) emerged as the top inhibited master molecules due to infection (Fig. 4).

Similar to NF-KB signaling, JAK/STAT signaling has been studied extensively as is critical for in that it converges diverse signals from the immune system to perform multiple functions, including resisting infection, maintaining immune tolerance, and enforcing innate barrier functions^50^,^51^. Indeed, our data show significant upregulation of JAK/STAT signaling and its associated genes/molecules, including IL-6, which implies an important role of this pathway in SA endophthalmitis. It has been shown that IL-6, a pleiotropic cytokine, plays a central role in the immune responses activated through the TNF-α and NF-κB pathways, as a first line of defense against infection in the acute-phase response^52^. Pathway enrichment analyses confirmed that the IL-6 signaling pathway is significantly enriched among temporal post-infection altered genes (Fig. 6). IL-6, upon receptor binding, activates STAT3 via the gp130/JAK/STAT pathway^53^,^54^. STAT3 is known to be important in the development of the immune system, in the maintenance of immune tolerance/privilege, and in active tumor surveillance^54^,^55^,^56^, among other cellular functions. Similar to IL-6, IL-10 confers broad anti-inflammatory properties, which can be amplified by a feed forward loop via the STAT3-induced expression of IL-10^57^,^58^. In the absence of inhibition by SOCS protein, the JAK/STAT pathway is also activated by IGF-1, another master regulator identified by our systems analysis. Given the important role of JAK/STAT signaling in autoimmune disease, cancer, and inflammatory diseases^51^,^59^, this pathway has become an attractive target for drug development. There are a wide variety of agents from the nonselective glucocorticoids to the selective JAK/STAT inhibitors with considerable therapeutic potential. Examples, including Ruxolitinib (JAK1 and JAK2 inhibitor), and tofacitinib (JAK3 inhibitor, approved for rheumatoid arthritis) are being evaluated in various clinical trials for psoriasis, inflammatory bowel disease, transplant rejection, and juvenile arthritis^60^. The availability of these FDA approved drugs targeting JAK/STAT signaling provides us the opportunity to test their efficacy in endophthalmitis or other ocular infections.

Many immune system specific transcription modulators are also temporally dysregulated post-infection. The CCAAT/enhancer-binding protein beta (C/EBPβ) has been shown to bind the IL-1 response element of the IL-6 gene and several other acute-phase response genes. C/EBPβ is a member of the C/EBP subfamily of leucine zipper containing transcription factors^61^,^62^ and is involved in increasing the expression of several target genes, including the pro-inflammatory cytokines IL-4 (T and B cell activation, proliferation, and differentiation)^63^, IL-5 (B cell activation)^64^, and IL-6 & TNF-α (initiation of acute phase response and regulation of innate and adaptive immunity)^65^,^66^. Interestingly, in addition to inflammation modulators, transcription factors, and cytokines, extracellular structural proteins, like SPP1, are also among the temporally dysregulated genes. The SPP1 gene codes for secreted phosphoprotein-1 (SPP1), more commonly known as osteopontin (OPN). OPN expression is increased in response to pro-inflammatory cytokines TNF-α and IL-1β, as well as other mediators of acute inflammation like angiotensin II, TGF-β, and parathyroid hormone (PTH)^67^. OPN, being a highly negatively charged extracellular structural protein, is capable of binding to integrin receptors on many cells types, including leukocytes^67^,^68^. Therefore, it has been suggested that OPN may play a role in immune cell adhesion, migration, and survival. Chemotactic response modulators like CSF1 are among the temporally dysregulated genes. The CSF1 gene codes for colony stimulating factor 1 (CSF1), also called macrophage colony stimulating factor (M-CSF). M-CSF acts locally within virtually all tissues of the body to increase the cellular activity of macrophages and the recruitment, and subsequent maturation and differentiation, of naïve monocytes to the site of infection and/or trauma^69^,^70^.

Another key master regulator identified in our study is IL-1β, a potent activator of immune responses directed against bacterial infections^71^. IL-1β mediates the migration of leukocytes, induces fever, and promotes the activation and polarization of T cells^72^. Compared to other inflammatory cytokines implicated in endophthalmitis, the expression and release of bioactive IL-1β are tightly regulated and require at least two distinct stimuli. The first is an inflammatory stimulus that results in the intracellular accumulation of pro-IL-1β, while the second stimulus activates a multiprotein complex, referred to as the “inflammasome”, which controls the Caspase-1 mediated cleavage of pro-IL-1β to active IL-1β^73^. Indeed, we have observed that the mouse retina and cultured microglia cells express components of the inflammasome (NLRP3, ASC and Caspase-1) and that SA challenge induces mRNA and protein expression of NLRP3 (unpublished, ARVO 2012 poster, Abstract # 2770). The counter regulation of IL-1β expression (Fig. 9) by Pam3 pretreatment and its reduced levels in TLR2 KO mice both imply a potential cross-talk between NLRP3 and TLR2 signaling, a current area of investigation in our laboratory. Similarly, the molecular determinants of inflammasome priming and activation in staphylococcal endophthalmitis need to be explored.

In summary, we present a comprehensive transcriptomic analysis of retinal gene expression at the early stages of staphylococcal endophthalmitis. Our systems biology analysis revealed that 13 hub genes, including the classical inflammatory genes (IL6, NFKB2, JUN, IL-1β, and CXCL2) which regulate retinal innate responses during infection. Moreover, Pam3 preconditioning dramatically altered the expression of these master regulators, suggesting that Pam3 is a potential candidate for the development of therapeutic modalities to prevent excessive inflammation and tissue damage in endophthalmitis. A thorough mining of our data on the basis of master regulators that are perturbed in response to infection demonstrated that key focus molecules are mostly involved in the innate immune response and that pretreatment of the host with innate immune activators increases resiliency and blunts immune system activation. Moreover, the identified key pathways can be targeted therapeutically to prevent or treat endophthalmitis.

## Materials and Methods

### Mice and Ethics Statement

C57BL/6 and TLR2 KO mice were purchased from the Jackson Laboratory (Bar Harbor, ME). The animals were housed in a restricted access DLAR facility at the Kresge Eye Institute, were maintained in a 12 h light to 12 h dark cycle, and fed LabDiet rodent chow (Labdiet Pico lab Laboratory, St Louis, MO) and water ad libitum. Female mice of 8–12 weeks of age were used throughout all experiments. The study was approved by the Institutional Animal Care and Use Committee (IACUC) of Wayne State University under protocol # A 08-02-13. All experimental procedures/methods were carried out in accordance with the approved guidelines of the Association for Research in Vision and Ophthalmology (ARVO) Statement for the Use of Animals in Ophthalmic and Vision Research.

### Induction of Endophthalmitis

The mice received intravitreal injections of 1.0 μl of PBS containing ~5,000 CFU of SA strain RN6390 (left eye) to induce endophthalmitis, as described previously^20^,^41^. The right eyes were left uninfected as a normal control. In the control group, the eyes were injected with sterile PBS. In the Pam3 treatment group, Pam3 (0.1 μg) was injected intravitreally. In the Pam3 pretreatment group, Pam3 (0.1 μg) was injected 24 h prior to SA injections. Following 3, 12, and 24 h infection, the mice were euthanized and the neuroretina was carefully removed without RPE or choroid and preserved in RNAlater^®^ solution (Ambion, Grand Island, NY) for downstream processing.

### Microarray

The mouse tissue gene expression profile was assessed at multiple time points (3 h, 12 h, 24 h) post-infection and PAM3 treatment using HT 430 2.0 PM Array plate (Affymetrix, Santa Clara, CA) containing >45,000 probes, allowing for the analysis of >30,000 well characterized mouse genes. Expression data were preprocessed using the Robust Multichip Average (RMA) method in R using the Bioconductor and associated packages^74^. Normalized high-quality arrays data were included in the unsupervised and supervised bioinformatics analysis. To identify statistically significant differences in gene expression, the microarray analysis was performed on at least 2–3 separate biological replicates at each time point.

### Unsupervised Analysis

The unsupervised analysis was performed using Principal Component Analysis and *Hierarchical* clustering to identify outliers and correlation among samples. Before PCA and Clustering, normalized data were preprocessed to remove transcripts with low expression for reducing false-positive results. Only transcripts with absolute expression ≥40 in at least 5% of the samples were included.

### Supervised analysis to identify differentially expressed genes and patterns

We used absolute fold changes (FC) and *p* values (student t-test) between control or vehicle treated samples and infected samples as the cut-off criteria to identify differentially expressed genes at each time point. The genes with an absolute fold change >1.5 and a *p* value <0.05 are considered to be differentially expressed. The fold change was calculated over multiple biological replicates to reduce false positive rates. It is well accepted in several studies that temporal analysis is the most effective strategy to identify the least false positive genes that are altered in a time-dependent manner to external stimulant using a low cutoff P value < 0.05^75^,^76^,^77^. To identify time and group dependent patterns from differentially expressed genes, we have adopted the Self Organizing Map (SOM) clustering technique^34^. We performed SOM clustering on transcript expression values using the Pearson correlation coefficient based distance metrics and a target of 40 groups. The analysis will help in identifying the genes depicting temporal expression changes (i.e., expression changes correlated with time of infection), as well as genes depicting expression changes only at early or late time points after infection. The genes depicting significant early, late, or temporal expression changes following SA infection will henceforth be collectively referred to as the SA infection signature.

### Counter Regulation Analysis

To identify genes that are involved in the treatment of infection, upon treatment or pre-treatment with PAM3, we performed counter regulation analysis on the transcripts from the SA infection signature that are differentially expressed due to SA infection. Counter regulation means that PAM3 treatment down-regulates genes that are up-regulated in SA infection and vice versa. SOM clustering of differentially expressed transcripts to 20 bins was performed to identify transcripts that are counter regulated by treatments. Bins of transcripts manifesting different degrees of counter regulation were identified and further filtered on the basis of magnitude and *p* value based significance of counter regulation. The final list of counter regulated genes was generated by considering genes that are counter regulated by a magnitude of fold change (FC) >1.2 compared to the degree of gene expression at 24 h after SA infection.

### Gene Ontology (GO) Analysis

To identify over-represented GO categories in differentially expressed genes from different comparisons, we used the Biological Processes and Molecular Functions Enrichment Analysis available from the Database for Annotation, Visualization and Integrated Discovery (DAVID)^78^. A *p*-value is assigned to each category on the basis of enrichments using jackknife Fisher exact probabilities.

### Pathway and Functional Analyses

Ingenuity Pathway Analysis (IPA) (Qiagen) was applied to early, late, or temporal expression changes due to SA infection and/or PAM3 treatments to identify biological pathways significantly over represented at the time of infection and counter regulated by PAM3 treatment. It calculates *p* values using Fisher’s exact test for each pathway and functions according to the fit of the user’s data to IPA databases. The pathways with *p*-values < 0.05 were considered significantly affected.

### Interactive Network Analysis

In addition to individual gene analysis, interactive network analysis was performed as the genes from different significantly affected pathways and functions crosstalk to each other, often resulting in some degree of pathophysiology. In the case of interactive networks, all of the identified genes were mapped to genetic networks available in the IPA database and were ranked by the score. The score (−log *p* value) is calculated using Fisher’s exact test and indicates the likelihood that a gene will be found in a network due to random chance.

### Regulator Module Analysis

The regulatory module analysis was used to identify the cascade of upstream transcriptional regulators involved in the observed gene expression changes to help identify key regulators (master regulators) and understand the underlying biological mechanisms. The analysis will help in identifying first which transcription regulators are significantly affected by the treatment, as well as determine whether they are activated or inhibited. The significance of overlap was determined using one-tailed Fisher’s exact test.

### RNA extraction and real time PCR analysis

Total RNA was extracted from retinal tissue using an RNeasy^®^ Mini Kit, as per the manufacturer’s instruction (Qiagen, Germantown, MD). Some RNA was used for the microarray analysis, and the remaining RNA was used for gene validation. cDNA was synthesized using 1.0 μg of total RNA using a Maxima first strand cDNA synthesis kit, as per the manufacturer’s instructions (Thermo scientific, Rockford, IL). Primers were purchased from Integrated DNA Technologies (Coralville, IA). PCR was performed using a StepOnePlus™ instrument (Applied Bio system, Grand Island, NY). The quantification of gene expression was determined via the comparative ΔΔCT method. The expressions in the test samples were normalized to endogenous reference GAPDH levels. All assays were performed in triplicate and repeated at least twice. The data are presented as the mean ± the SD and statistical analysis were performed using one-way analysis of variance (ANOVA).

### ELISA, Western Blotting, Histopathology, and Immunostaining

The detailed methods for validation of target molecules using ELISA, Western Blotting, and Histopathology and Immunostaining are provided in our previous studies^3^,^19^,^31^,^32^,^35^.

## Additional Information

**How to cite this article**: Rajamani, D. *et al.* Temporal retinal transcriptome and systems biology analysis identifies key pathways and hub genes in *Staphylococcus aureus* endophthalmitis. *Sci. Rep.* **6**, 21502; doi: 10.1038/srep21502 (2016).

## Supplementary Material

Supplementary Information

## Figures and Tables

**Table 1:** Top signaling pathways and their associated molecules.

Ingenuity canonical pathways		Molecules
Constitutive	Estrogen Receptor Signaling	TAF4B, THRAP3, TAF5, MED1, MED13L, MED4, TAF15
	CD27 Signaling in Lymphocytes	MAP3K12, MAP2K7, IKBKG, IKBKAP
	TNFR1 Signaling	IKBKG, MADD, XIAP
	Protein Ubiquitination Pathway	DNAJC28, DNAJC24, UBR1, ANAPC5, UBE3A, XIAP, SKP2, DNAJC11
	AMPK Signaling	CAB39, PPP2R3A, STRADA, PPP2R2B, RPTOR
Temporal	IL-17A Signaling in Fibroblasts	FOS, JUN, NFKBIA, LCN2, CEBPD, CEBPB, NFKB2, IL6, NFKBIZ, MAPK11
	IL-10 Signaling	IL33, FOS, IL4R, JUN, NFKBIA, IL1B, STAT3, IL1R1, NFKB2,IL6, FCGR2B, MAPK11
	IL-6 Signaling	IL6R, STAT3, IL1R1, CEBPB, NFKB2, IL6, MAPK11, IL33, FOS, NFKBIA, JUN,PIK3C3,IL1B,ATM,MCL1
	JAK/Stat Signaling	FOS, PIK3C3, PIAS1, CDKN1A, CISH, PTPN1, SOCS2, STAT3, IL6, STAT1, ATM
	IL-9 Signaling	PIK3C3, CISH, SOCS2, BCL3, STAT3, NFKB2, STAT1, ATM
