# Supplementary material for: Temporal retinal transcriptome and systems biology analysis identifies key pathways and hub genes in Staphylococcus aureus endophthalmitis
Source: Sci Rep. 2016 Feb 11;6:21502. doi: 10.1038/srep21502 (PMC4749995; doi:10.1038/srep21502)
Supplement: Supplementary Information [file srep21502-s1.pdf]

Supplementary Document

Temporal retinal transcriptome and systems biology analysis identifies key pathways and hub genes in *Staphylococcus aureus* endophthalmitis

Authors:

Deepa Rajamani[4†], Pawan Kumar Singh[1,2†], Bruce G. Rottmann[2], Natasha Singh[3], Manoj K. Bhasin[3] , and Ashok Kumar [1, 2, 3*]

† First author/equal contribution

Affiliations:

[1] Kresge Eye Institute, Wayne State University, Detroit, MI

[2] Department of Anatomy and Cell Biology, Wayne State University, Detroit, MI

[3] Department of Immunology & Microbiology, Wayne State University, Detroit, MI

[4] BIDMC Genomics, Proteomics, Bioinformatics and Systems Biology Center, Harvard Medical School, Boston, MA

***Corresponding Author**

Ashok Kumar, Ph.D.
E-mail: akuma@med.wayne.edu

Manoj K. Bhasin, Ph.D.
E-mail: mbhasin@bidmc.harvard.edu

Supplementary Figure Legends:

Figure S1. Differentially expressed genes identified from supervised analyses of SA post-infection data. A. Venn Diagram comparing significantly differentially expressed genes identified from the following comparisons: controls vs. 3h post-infection, controls vs. 12h post-infection, and controls vs. 24h post-infection. The genes were selected using supervised analysis on the basis Absolute Fold Change (AFC) and p value (AFC >1.5, p <0.05) by pairwise comparison of the groups. The analysis was performed on preprocessed data by filtering low-expressing probes on the basis of absolute intensity (intensity <40 in all samples). **B.** Heatmap of 1,434 genes commonly differentially expressed among different post-infection time points compared to controls**.** Columns represent the samples and rows represent genes. Gene expression levels are shown as a pseudocolor scale (-1 to 1) with red denoting a high expression level and green denoting a low expression level. **C.** GO clusters of biological processes and metabolic functions significantly enriched in 1,434 genes commonly differentially expressed among different post-infection time points compared to controls. A GO enrichment score of 1.3 is equivalent to linear p value of 0.05.

Figure S2: Functional and Gene Ontology Enrichment analysis of constitutively and temporally altered genes post-infection. GO clusters of biological processes and metabolic functions significantly enriched in constitutive **(A)** and temporal **(B)** post-infection altered genes. Each bar represents a significantly enriched GO cluster, with significance shown as enrichment score on x-axis (Enrichment Score = -log p value). **C)** Cologram of functional categories and disease that are significantly linked to temporal post-infection altered genes.

Figure S3: Transcriptional profile of PAM3 treated samples (PAM3) and PAM3 pre-treated and SA infected samples (PAM3+SA). A) Venn Diagram comparing significantly differentially expressed genes identified only in PAM3 treated samples without infection, or post-infection in PAM3 pre-treated samples. The analysis identified 85 commonly differentially expressed genes. **B)** Heatmap of 85 genes commonly differentially expressed in PAM3 treated and PAM3+SA samples. Columns represent samples and rows represent genes. Gene expression levels are shown on a pseudocolor scale (-1 to 1), with red denoting high expression levels and green

denoting low expression levels. **C)** Functional and disease enrichment analysis of genes that are commonly differentially expressed in PAM3 treated and PAM3+SA samples**.** Each bar represents a significantly enriched functional category. **D)** Pathway enrichment analysis of genes that are commonly differentially expressed in PAM3 treated and PAM3+SA samples**.**

Figure S4: qRT-PCR analysis of regulatory molecules and their correlation with gene expression data. C57BL/6 mice were intravitreally injected with S. aureus (SA) or Pam3Cys (either alone or 24h pre-infection) for the indicated time points. Total RNA was extracted, reverse transcribed, and subjected to qRT-PCR using specific primers for thirteen master regulators (IGF1, Jun, STAT3, NUPR1, CEBPB, CSF1, CyR61, EGFR1, SPP1, TGM2, IL-6, IL-1β, and CXCL2) genes, with glyceraldehyde 3-phosphate dehydrogenase (GAPDH) as the control. Modulations of gene expression were expressed as relative fold changes with respect to the GAPDH control. Statistical analysis was performed using one-way ANOVA for comparisons of control versus stimulated cells over time. Data points and bars represent the mean ± the SD of triplicates from three independent experiments.

Supplementary Figures:

Figure S1

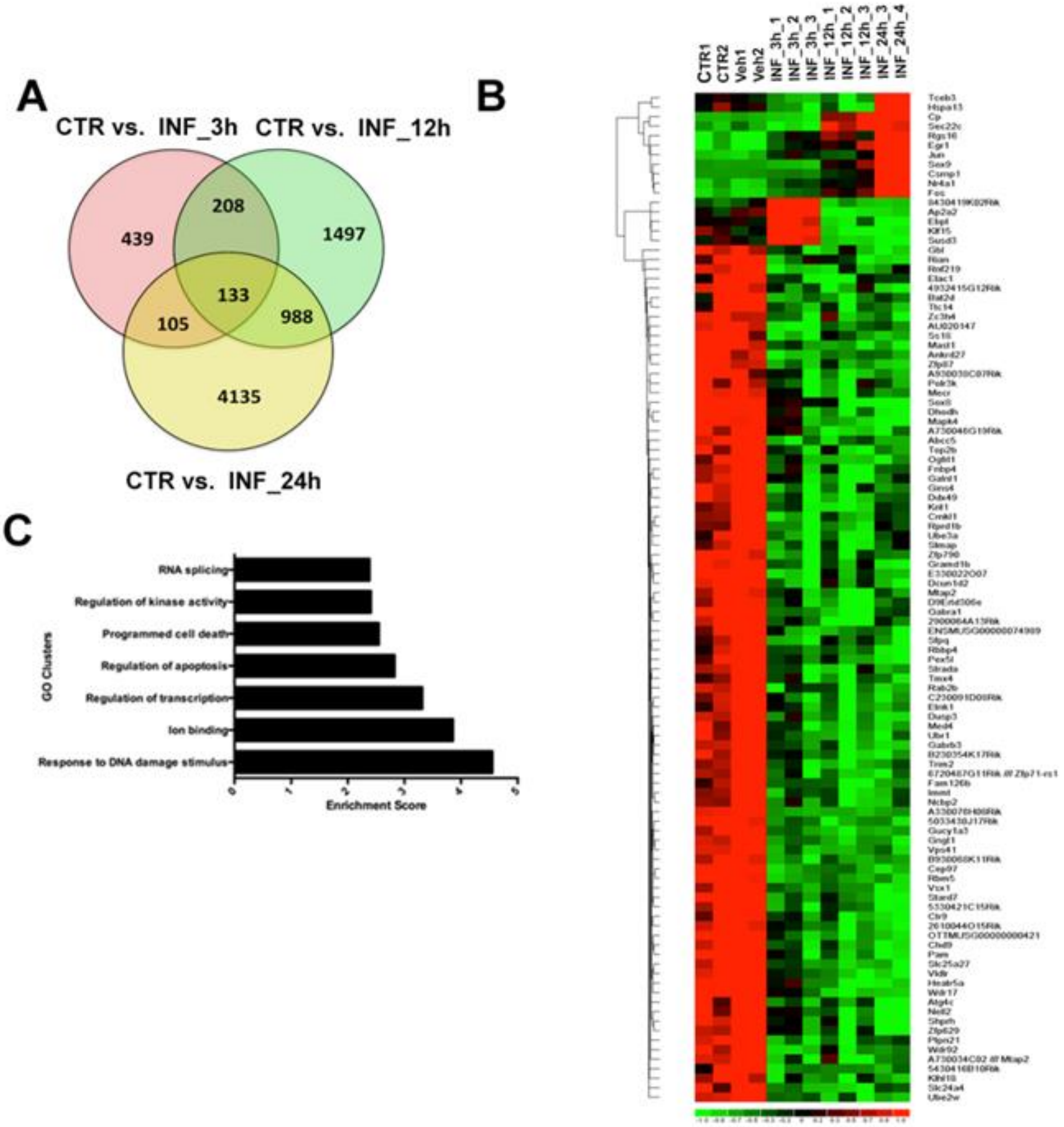

Figure S2

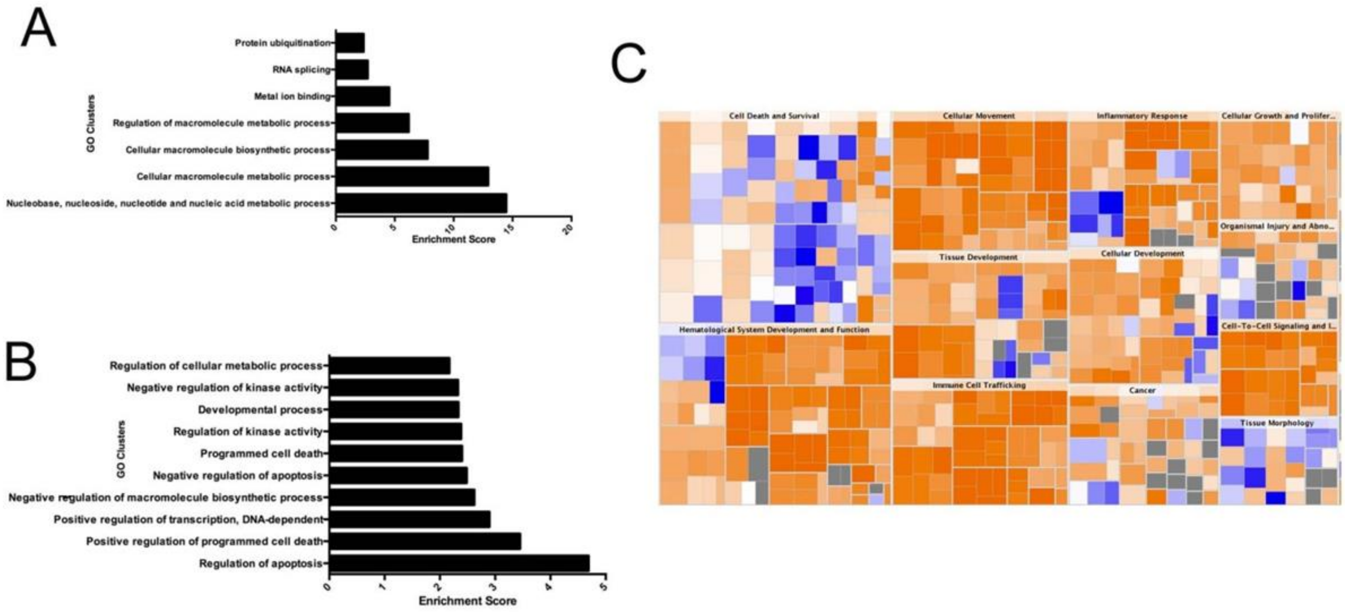

A
Protein ubiquitination
RNA splicing
Metal ion binding
Regulation of macromolecule metabolic process
Cellular macromolecule biosynthetic process
Cellular macromolecule metabolic process
Nucleobase, nucleoside, nucleotide and nucleic acid metabolic process
GO Clusters
0
5
10
15
20
Enrichment Score
B
Regulation of cellular metabolic process
Negative regulation of kinase activity
Developmental process
Regulation of kinase activity
Programmed cell death
Negative regulation of apoptosis
Negative regulation of macromolecule biosynthetic process
Positive regulation of transcription, DNA-dependent
Positive regulation of programmed cell death
Regulation of apoptosis
GO Clusters
0
1
2
3
4
5
Enrichment Score
C
Cell Death and Survival
Cellular Movement
Inflammatory Response
Cellular Growth and Prolifer...
Organismal Injury and Abno...
Tissue Development
Cellular Development
Hematological System Development and Function
Cell-To-Cell Signaling and I...
Immune Cell Trafficking
Cancer
Tissue Morphology

Figure S3

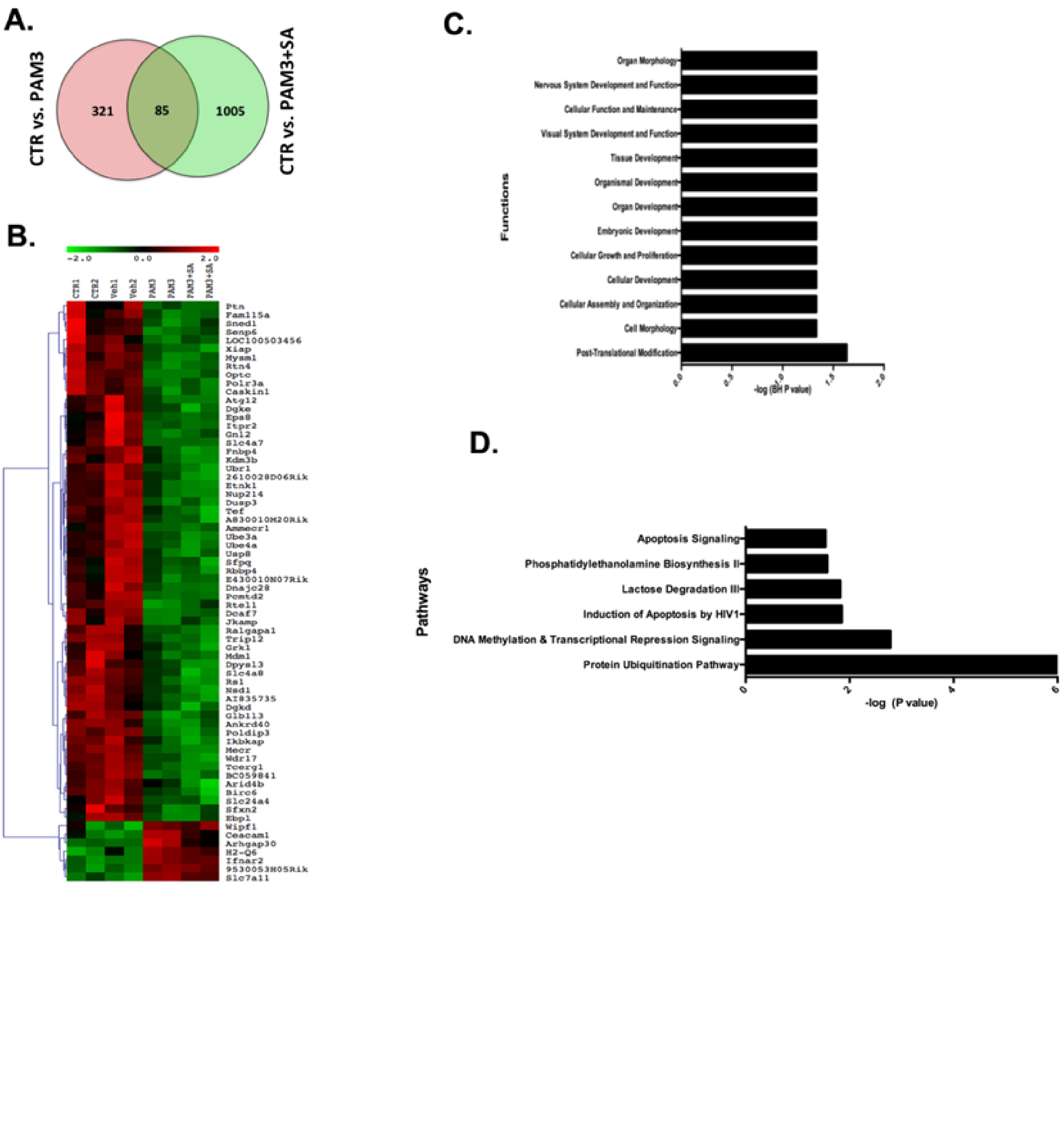
A.
CTR vs. PAM3
CTR vs. PAM3+SA
321
85
1005
B.
-2.0
0.0
2.0
CTR1
CTR2
Veh1
Veh2
PAM3
PAM3
PAM3+SA
PAM3+SA
Ptn
Fam115a
Sned1
Senp6
LOC100503456
Xiap
Mysm1
Rtn4
Optc
Polr3a
Caskin1
Atg12
Dgke
Eps8
Itpr2
Gnl2
Slc4a7
Fnbp4
Kdm3b
Ubr1
2610028D06Rik
Etnk1
Nup214
Dusp3
Tef
A830010M20Rik
Ammecr1
Ube3a
Ube4a
Usp8
Sfpq
Rbbp4
E430010N07Rik
Dnajc28
Pcmtd2
Rtel1
Dcaf7
Jkamp
Ralgapa1
Trip12
Grk1
Mdm1
Dpysl3
Slc4a8
Ral
Nsd1
AI835735
Dgkd
Glb1l3
Ankrd40
Poldip3
Ikbkap
Mecr
Wdr17
Tcerg1
BC059841
Arid4b
Birc6
Slc24a4
Sfxn2
Ebp1
Wipf1
Ceacam1
Arhgap30
H2-Q6
Ifnar2
9530053H05Rik
Slc7a11
C.
Functions
Organ Morphology
Nervous System Development and Function
Cellular Function and Maintenance
Visual System Development and Function
Tissue Development
Organismal Development
Organ Development
Embryonic Development
Cellular Growth and Proliferation
Cellular Development
Cellular Assembly and Organization
Cell Morphology
Post-Translational Modification
0.0
0.5
1.0
1.5
2.0
-log (BH P value)
D.
Pathways
Apoptosis Signaling
Phosphatidylethanolamine Biosynthesis II
Lactose Degradation III
Induction of Apoptosis by HIV1
DNA Methylation & Transcriptional Repression Signaling
Protein Ubiquitination Pathway
0
2
4
6
-log (P value)

Figure S4

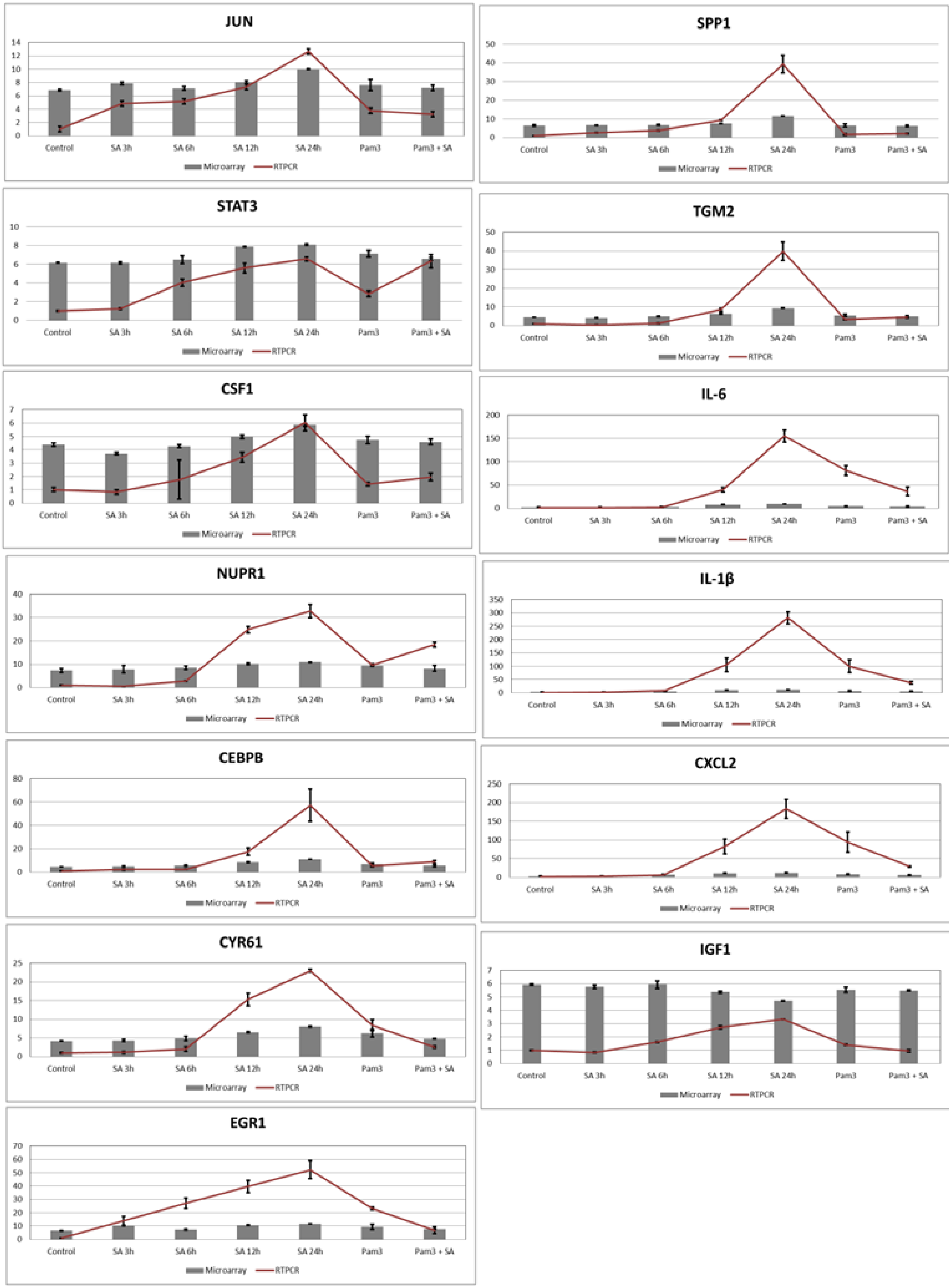

JUN
SPP1
STAT3
TGM2
CSF1
IL-6
NUPR1
IL-1β
CEBPB
CXCL2
CYR61
IGF1
EGR1
Control
SA 3h
SA 6h
SA 12h
SA 24h
Pam3
Pam3 + SA
Microarray
RTPCR
